# Bioaccumulation and Health Risk Assessment of Trace Elements in *Oreochromis niloticus* in Bukit Merah Lake, Malaysia

**DOI:** 10.21315/tlsr2022.33.2.9

**Published:** 2022-07-15

**Authors:** Mohd Ilman Che Abdullah, Amir Shah Ruddin Md Shah, Hazzeman Haris

**Affiliations:** School of Biological Sciences, Universiti Sains Malaysia, 11800 USM Pulau Pinang, Malaysia

**Keywords:** Carcinogenic Risk, Non-Carcinogenic Risk, Fish, Human Health Risks, Trace Elements, Risiko Karsinogenik, Risiko Bukan Karsinogenik, Ikan, Risiko Kesihatan Manusia, Unsur Surih

## Abstract

This study aims to determine the level of nine trace elements – As, Cd, Cr, Cu, Fe, Mn, Ni, Pb and Zn in liver, gill and muscle of *Oreochromis niloticus* in Bukit Merah Lake (BML). The concentration of trace elements was measured using Inductively Coupled Plasma-Optical Emission Spectrometers (ICP-OES). Cd and Ni were found below the detection level, while Cr was only detected in gill and muscle tissues. The Metal Pollution Index (MPI) established were liver > gill > muscle. The ranking order of trace elements in the gills was Fe > Zn > Mn > Pb > As > Cu > Cr. For the liver, the ranking order was Fe > Cu > Zn > As > Mn > Pb, while in the muscle, the ranking order was Fe > Zn > Pb > As > Cu > Mn > Cr. The estimated daily intake (EDI) for all the trace elements in this study was calculated based on 168 g.day^−1^ of Malaysians’ fish consumption, indicating no potential risk. From the human health point of view, there was no significant non-carcinogenic risk of individual trace elements as evaluated by Target Hazard Quotient (THQ). As indicated by the Hazardous Index (HI), the cumulative effect of all trace elements also suggested no potential of non-carcinogenic risk. The carcinogenic risks assessed from Pb and As were also neglectable and there was no likelihood of getting cancer during one’s life span.

## Abstract (ms)

Kajian ini bertujuan untuk menentukan sembilan unsur surih – As, Cd, Cr, Cu, Fe, Mn, Ni, Pb dan Zn di dalam hati, insang dan otot *Oreochromis niloticus* di Tasik Bukit Merah. Kepekatan unsur surih diukur dengan menggunakan instrumen plasma gandingan teraruh – spektrometer emisi optik (ICP-OES). Unsur surih Cd dan Ni di dapati di bawah paras pengesanan, manakala Cr hanya dikesan di dalam insang dan tisu otot. Indeks pencemaran logam (MPI) menunjukkan bahawa hati > insang > otot. Turutan susunan unsur surih di dalam insang adalah Fe > Zn > Mn > Pb > As > Cu > Cr. Bagi hati, turutan susunan adalah Fe > Cu > Zn > As > Mn > Pb, manakala di dalam otot turutan susunan adalah Fe > Zn > Pb > As > Cu > Mn > Cr. Anggaran pengambilan harian (EDI) bagi kesemua unsur surih di dalam kajian ini dikira berdasarkan 168 g.hari^−1^ pemakanan ikan oleh penduduk Malaysia, menunjukkan tiadanya potensi risiko. Berdasarkan sisi kesihatan manusia, tidak terdapat risiko bukan karsinogenik yang signifikan bagi setiap unsur surih seperti yang dinilai oleh darjah bahaya sasaran (THQ). Kesan kumulatif bagi kesemua unsur surih seperti yang ditunjukkan oleh indeks bahaya (HI) juga mencadangkan tiadanya potensi risiko bukan karsinogenik. Risiko karsinogenik dinilai berasaskan risiko karsinogenik (CR) yang disebabkan oleh Pb dan As adalah boleh diabaikan dan tiada kemungkinan untuk mendapat barah dalam jangka hayat seseorang.

**Highlights**

- Trace elements.
- Carcinogenic and non-carcinogenic risks.
- Health risk assessment.

## INTRODUCTION

Trace elements are frequently highlighted due to their toxicity, bioaccumulation, non-degradability, persistence and biomagnification at various trophic levels in the aquatic ecosystems (Kortei *et al*. 2020; Yu *et al*. 2020). Ongoing deposition of trace elements in the lake ecosystem through natural (e.g., weathering, erosion) and anthropogenic activities (e.g., domestic waste, deforestation, usage of chemical fertiliser and pesticide) in recent decades increased the level of trace elements uptake by aquatic organisms such as fish. Fish stand at a higher trophic level and may accumulate a high level of trace elements via either gills or diet (alimentary tract). This has raised concern because fish consumption in Malaysia is high at an estimated 168 g.day^−1^, which is considered only second to that of Japan (Nurul Izzah *et al*. 2016). Fish has long been regarded as an affordable source of high-quality protein. Fish contains high level of polyunsaturated omega-3 fatty acids (PUFAs), eicosapentaenoic acid (EPA), docosahexaenoic acid (DHA), and other essential amino acids (Ullah *et al*. 2017; Annette *et al*. 2018).

However, high level of trace elements in fish might counter the benefits of fish consumption. The trace elements can be grouped into essential (Fe, Cu, Mn, Zn, etc.) and potentially toxic (As, Pb, Cd, Hg, etc.). Nevertheless, all the trace elements can pose a serious health threat when exceeding the permissible limit regardless of their classification. Several health risks are associated with the consumption of fish that are contaminated with trace elements, including neurological effects, lung fibrosis, various types of cancer, defective bone mineralisation, renal failure and reproductive problems (Bosch *et al*. 2016; Titilawo *et al*. 2018; Mwakalapa *et al*. 2019; Alipour *et al*. 2021).

*Oreochromis niloticus* (Nile tilapia) is one of the economic importance of freshwater fish species in Malaysia. Since its introduction to Malaysia, *O. niloticus* has been regarded as the second most important fish species for freshwater fish farming after catfish (*Clarias macrocephalus*) (Mohammed *et al*. 2016; Ishak *et al*. 2020). The production of *O. niloticus* in Malaysia is estimated at 32,526.44 metric tons with a wholesale value of MYR299.7 million in 2017 (Malaysian Annual Fisheries Statistics 2017), as cited in Mohammad Ridzuan *et al*. (2020). Due to its commercial value and high consumption rate among the local population, it is of paramount importance to ensure the safety level for human consumption. Currently, there is no report yet on the assessment of trace elements in *O. niloticus* in Bukit Merah Lake (BML). Thus, this study was designed to evaluate the trace elements (As, Fe, Cr, Cu, Cd, Mn, Ni, Pb, Zn) concentration in three different parts (gill, muscle and liver) of *O. niloticus*. At the same time, the health risk arising from the consumption of the edible parts (muscle) of this fish was also assessed. This preliminary study will provide useful information for constant monitoring and health assessment in the future for this fish species in BML.

## MATERIALS AND METHODS

### Site Description

**Figure 1:**
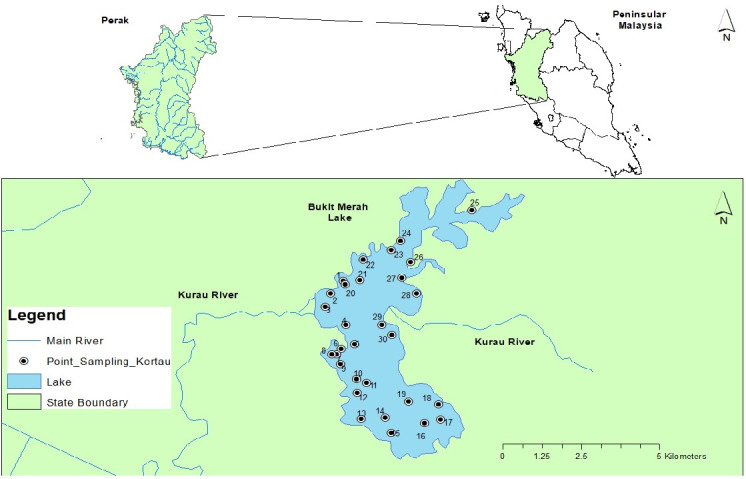
The location of Bukit Merah Lake (BML) in Peninsular Malaysia.

The construction of Bukit Merah Lake (BML) was completed in 1906 using a modified homogeneous embankment that was constructed in the upper stream of the Kurau River and Merah River confluence. This 41 km^2^ lake is located in the district of Kerian in the northern Perak at longitude of 5° 2′ 00″ and latitude of 100° 40′ 00″, and is divided into north and south lakes by a 4.7 km railway track (Fig. 1). BML is the oldest human-made lake in Peninsular Malaysia. This lake provides much-needed water for a double cropping system of paddy fields under the Kerian Irrigation Scheme, which covers 24,000 hectares of paddy fields. Currently, BML also supplies freshwater for domestic and commercial demand in both Kerian and Larut Matang districts, with an estimated 200,000 residents. However, along with its long history, this lake has suffered from various anthropogenic activities (domestic waste, sand mining, agriculture and deforestation), which increases the influx of contaminants containing trace elements into the lake ecosystem (Fadhullah *et al*. 2020; Hidzrami 2010).

### Sample Collection

The *O. niloticus* was collected on 11 April 2018 by purchasing it from the local fisherman in BML. The fish was washed with deionised water and identification was conducted based on the guideline listed by Kottelat (2013). All the fish samples were kept frozen at −20°C prior to analyses. Five individuals of *O. niloticus* were used for the determination of trace elements. The total length of *O. niloticus* used in this study ranged from 18.3 cm to 20.5 cm and the weight ranged from 134.78 g to 188.29 g.

### Digestion Method

Prior to the experiment, all the non-steel apparatus were sterilised by soaking them overnight in a diluted 10% nitric acid and rinsed with deionised water. Before the sample preparation, the fish maturity stage was determined by examining the fish’s total length as proposed by Mohsin and Ambak (1991). In this study, only matured fish were used to assess the trace elements. The boneless muscle (above the lateral line and between the dorsal part and the caudal fins) was removed using a sterilised stainless-steel scalpel. Five individuals of the same size were used. Each of the samples (gill, muscle and liver) was homogenised and separated into two equal parts for the duplicate procedure. The samples were oven-dried in an oven at 80°C until all the samples reached the constant weight and were left to cool in the desiccator (Fathi Alhashmi *et al*. 2012; Ahmad & Sarah 2015).

The samples were crushed into a fine powder using a porcelain mortar and pestle which was pre-washed with 10% nitric acid (HNO_3_). The digestion methods suggested by Radojevic and Bashkin (2006) and EPA Method 3051A (EPA 2007) were used in this study. The aqua regia solution was prepared by mixing 150 mL of HCI solution (130 mL concentrated HCI + 120 mL of mili-Q water) with 50 mL of concentrated HNO_3_. The fish samples were placed in the digestion tube and immersed overnight in 5 mL of aqua regia solution. Then the samples were digested in duplicate using a microwave digester. The temperature was set at 180°C for 9.5 min and allowed to cool at room temperature in the microwave. The digested samples were filtered with a 0.45 μm Whatman filter paper into a 50 mL volumetric flask. The total of 0.25 M HNO_3_ was added up to the mark before analysed using Perkin Elmer Optima 5300 DV ICP-OES for the presence of trace elements. The trace elements content were determined based on dry weight (mg.kg^−1^ d.w) and later converted into wet weight (mg.kg^−1^ w.w) using moisture content for each sample. The precision of the analyses was assessed using certified reference material of fish protein (DORM 4). The recovery rates were ranged from 89.4% to 107%, thus confirming the accuracy of the analyses (Table 1).

### Metal Pollution Index (MPI)

The MPI is an index used to compare the total trace elements accumulated in various tissues (muscle, liver, gonad, kidney and gill) in fish (Łuczyńska *et al*. 2018; Khalil *et al*. 2020; El-Gaar *et al*. 2021). This index provides a direct result for easy comparison of accumulation across different organs or species. The MPI value > 2 is considered as not impacted; 2 < MPI < 5 is considered as very low contamination and 5 < MPI < 10 is considered as low contamination. This index is calculated according to the equation below:


$$\text{MPI }(\text{mg}.{\text{kg}}^{-1})={\left({\text{C}}_{\text{f}1}\times {\text{C}}_{\text{f}2}\times {\text{C}}_{\text{f}3}\times {\text{C}}_{\text{fn}}\right)}^{1/\text{n}}$$

Where:

C_f1_ = concentration of the first metal
C_f2_ = concentration of the second metal
C_f3_ = concentration of the third metal

### Estimated Daily Intake (EDI)

The EDI of the trace elements was calculated based on the equation below (Ashraf *et al*. 2012):


EDI=C×IR/BW

Where:

C = mean concentration of heavy metals in foodstuff (mg.kg^−1^)
IR = daily ingestion rate in Malaysia = 168 g.day^−1^ (Nurul Izzah *et al*. 2016)
BW = body weight (64 kg for Malaysian adults) (Anual *et al*. 2018)

### Target Hazard Quotient (THQ)

The THQ is the estimation of the non-carcinogenic level due to the exposure of the pollutants. If the value of THQ is below 1, it means that there is no possible health threat, whereas if the value of THQ is ≥ 1, it means that there is a possible health threat and that corrective measures should be taken (Kortei *et al*. 2020). THQ index can be calculated using the equation below:


$$\text{THQ}=(\text{EFr}\times \text{ED}\times \text{FiR}\times \text{C}/\text{RfD}\times \text{BW}\times \text{TA})\times 10^{-3}$$

Where:

EFr = the total exposure frequency, which is equivalent to 365 days.year^−1^
ED = the exposure duration (75 years)
FiR = the rate of fish ingestion (g.day^−1^)
C = the mean concentration of trace elements in foodstuff (mg.kg^−1^)
RfD = the oral reference dose according to USEPA
BW = the mean of body weight (64 kg)
TA = the mean of exposure time (365 days.year^−1^ × ED)

The oral reference dose (RfD) for the trace elements As is 0.0003 mg.kg^−1^.day^−1^, Cd is 0.0001 mg.kg^−1^.day^−1^, Cr is 1.5 mg.kg^−1^.day^−1^, Cu is 0.04 mg.kg^−1^. day^−1^,Fe is 0.7 mg.kg^−1^.day^−1^, Mn is 0.014 mg.kg^−1^.day^−1^, Ni is 0.02 mg.kg^−1^.day^−1^, Pb is 0.0035 mg.kg^−1^.day^−1^ and Zn is 0.3 mg.kg^−1^.day^−1^.

### Hazardous Index (HI)

The hazardous index (HI) is summative of the THQ for all trace elements to which an individual might be exposed. The HI was calculated using the equation below (Łuczyńska *et al*. 2018). The value of HI ≥ 1, reveals the possible non-carcinogenic risk to humans.


HI=Σ THQ=THQ (As)+THQ (Cr)+THQ (Cu)+THQ (Fe)+THQ (Mn)+THQ (Pb)+THQ (Zn)

### Carcinogenic Risk (CR)

This index assesses the probability of an individual developing cancer over the lifespan due to exposure to carcinogenic metal(loid)s. Assessment of CR was conducted on Pb and As only where the cancer slope factors (CSF) for Pb and As were 0.0085 mg.kg^−1^ day^−1^ and 1.5 mg.kg^−1^.day^−1^, respectively (Ullah *et al*. 2017). However, the calculation of carcinogenic risk should be based on the inorganic As (_i_As), which is known to be more toxic (Varol & Sünbül 2018; Shakeri *et al*. 2020; Fakhri & Sarafraz 2021). Although the analysis for the _i_As was not conducted this study, the carcinogenic risk calculation assumed that _i_As in the fish ranged from 1% to 10% of the total As (Zhong *et al*. 2018; Töre *et al*. 2021). Both authors used the maximum assumption percentage of _i_As. Therefore, in this study, the _i_As was assumed to be 10% of the total As. According to USEPA (2018), the value of CR ≤ 10^−6^ is considered an acceptable range, whereas the value of CR is ≥ 10^−3^ is considered intolerable. The calculation of CR was given by the following equation:


CR=CSF×EDI

Where:

CSF = Cancer slope factor by USEPA (mg.kg^−1^.day^−1^)
EDI = Estimation Daily Intake

## RESULTS

The concentrations of trace elements in three different parts of *O. niloticus* were significantly differents (*P* < 0.05), as shown in Table 2. The MPI values for the total trace elements in different parts of the fish were liver (3.10) > gill (0.19) > muscle (0.10).

The ranking order of trace elements in the gills was Fe > Zn > Mn > Pb > As > Cu > Cr. The ranking order for the liver was Fe > Cu > Zn > As > Mn > Pb while for the muscle was Fe > Zn > Pb > As > Cu > Mn > Cr. However, Cd and Ni in all tissues (muscle, gills and liver) were recorded below the detection level. The lowest concentrations of trace elements were recorded in the muscle tissue, which correlated with the results of MPI.

The concentration of trace elements in the edible part (muscle) was used to assess both non-carcinogenic and carcinogenic health risks. Table 3 shows the value of EDI, THQ, and HI. All the EDI were found to be below the recommended value for all trace elements. The order of THQ for each trace element was Pb (78.25%) > _i_As (9.13%) >Zn (4.84%) > Cu (4.11%) > Fe (2.85%) > Mn (0.78%) > Cr (0.04%). Nevertheless, the value of THQ for each trace element was found to be below 1, which indicated a single trace element posed no potential non-carcinogenic risk. The cumulative effect of THQ, as represented by the HI index was also found to be lower than 1, which reflected no possibility of non-carcinogenic risk to the local population due to the combined effect of the trace elements. This study established two non-essential trace elements: Pb and _i_As accounted for 87.38% of the total THQ as presented by the HI. The carcinogenic risk has only been reported for _i_As and Pb as the cancer slope factors were not available for the other trace elements. The values of CR for _i_As and Pb in this study were 3.94 × 10^−6^ and 2.23 × 10^−6^, respectively.

## DISCUSSION

This study revealed the selective bioaccumulation of *O. niloticus* across different tissues, which depended on several factors such as the affinity of trace elements to bind with different molecular groups within the cells of respective organs, the uptake and removal rates of trace elements, the intensity of fish metabolism and the physiological roles of each organ (El-Sadaawy *et al*. 2013; Aissaoui *et al*. 2017). The MPI values in the gills and muscle of *O. niloticus* in BML were classified as not impacted, whereas the MPI in the liver was classified as very low contamination. The trace elements found in different organs of *O. niloticus* was consistent with many previous reports (El-Sadaawy *et al*. 2013; Taweel *et al*. 2013b; Abdel-Khalek *et al*. 2016; Ju *et al*. 2017; El-Gaar *et al*. 2021). The liver accumulated the highest level of all the essential trace elements, which is typically related to the presence of the cysteine-rich binding protein known as metallothioneins in the hepatic cells. Liver acts as a reservoir for the essential trace elements, such as Fe, Cu and Zn. These three essential trace elements play essential roles as cofactors and coenzymes in many enzymatic metabolisms in the liver. At the same time, the accumulation of non-essential trace elements, such as Pb and As, was also high. This is due to the liver’s function as a major homeostasis organ in which processes such as toxicant bioaccumulation, transformation, storage and detoxification occur (Maurya & Malik 2018).

The high concentration of trace elements found in the gills after the liver is because the gills are the first organ that comes into contact with the trace elements in the water column. The gills have a large surface area with highly vascularised blood capillaries, which increases the absorption and the accumulation of trace metal elements (Abdel-Khalek *et al*. 2016). Lower concentrations of trace elements in the muscle tissue are widely acknowledged and reported in the literature (Maurya & Malik 2018; Lobos *et al*. 2019; Mwakalapa *et al*. 2019; Kindie *et al*. 2020). The muscle is not a metabolically active tissue and thus, is less active in accumulating trace elements. Additionally, the muscle tissue is protected by a layer of skin that prevents direct contact with the trace elements (Dwivedi *et al*. 2015). Findings from this study corroborates the previous studies on *O. niloticus* (El-Moselhy *et al*. 2014; Rosli *et al*. 2018; Morshdy *et al*. 2021; Rizk *et al*. 2022).

Unlike in the muscle and the gill, Cr was not detected in the liver of the fish. The possible explanation for this is that Cr tends to accumulate in the gill compared to the liver when the concentration of Cr is low (Ciftci *et al*. 2010). The reported value of Cr in the water of BML is 8.5 × 10^−5^ mg.L^−1^ (Shuhaimi-Othman *et al*. 2010). This value is considered low based on the National Water Quality Standards for Malaysia (NWQS) (5.0 × 10^−2^ mg.L^−1^) and thus, explains the absence of Cr in the liver of *O. niloticus*.

As can have acute and chronic effects in humans, such as neurotoxicity, skin problems, cardiovascular disease, hematological, respiratory symptoms, and various types of cancer (Bosch *et al*. 2016; Rasheed *et al*. 2016; Sanyal *et al*. 2017). The oral RfD for inorganic As is 0.0003 mg.kg^−1^.day^−1^. Higher level will cause dermatitis, formation of liver carcinoma, and reduced neuron transmission (Korkmaz *et al*. 2017). The concentration of As in the muscle tissue of *O. niloticus* in this study was lower than the reported values of muscle tissue in *O. niloticus* from Cempaka Lake, Selangor, which ranged from 1.79 mg.kg^−1^–6.26 mg.kg^−1^ (Taweel *et al*. 2013a) and *O. niloticus* from aquaculture sites in Jelebu, Negeri Sembilan which ranged from 0.41 mg.kg^−1^–1.07 mg.kg^−1^ (Kah *et al*. 2015).

Many physiological processes such as hemoglobin synthesis enzymatic reaction, metabolism, and immune system, require the presence of a small amount of Cu (Azaman *et al*. 2015; Rodríguez *et al*. 2017). However, high level of Cu can cause toxicity to human when exceeding the standard level and cause neurotoxicity symptoms known as Wilson’s disease, oxidative stress and various cellular abnormalities (Resma *et al*. 2020; Storelli *et al*. 2020). The average Cu concentration reported in the literature for fish muscle in Malaysia ranged from 0.01 mg.kg^−1^–2.13 mg.kg^−1^ (Taweel *et al*. 2013b; Yaakub *et al*. 2017), and the level of Cu reported in this study was within the range and below the permissible limit set by the Malaysian Food Act (1983) and Regulations (1985) at 30 mg.kg^−1^.

Cr is a non-essential trace element that does not usually cause toxicity to living organisms. However, at higher concentration, chromium is reported to have carcinogenic effects that can lead to liver and kidney cancers. Exposure to Cr is also associated with respiratory problems (Chiesa *et al*. 2018). According to the Malaysian Food Act (1983) and Regulations (1985) and WHO (1985), the permissible limit of Cr is 0.2 mg.kg^−1^ and 0.6 mg.kg^−1^, respectively. Based on these levels, the level of Cr in *O. niloticus* in BML was below the permissible limit.

Fe recorded the highest concentration of trace elements in all parts of the fish. Fe plays a vital role in respiratory pigments, such as hemoglobin and myoglobin, and also acts as a cofactor in many enzymatic reactions. The most prominent sign of Fe deficiency is anemia, weakness and loss of focus. The level of Fe recorded in all parts of the fish exceeded the permissible limit set by the Malaysian Food Act (1983) and Regulations (1985) at 0.5 mg.kg^−1^. However, according to WHO (1985), the level of Fe in this study was still within the permissible level of 50 mg.kg^−1^. Many findings in this country reported that Fe comprised the highest trace element in fish (Widad & Abdullah 2013; Rosli *et al*. 2018). According to Shuhaimi-Othman *et al*. (2010), the concentration of Fe in the water column of BML was recorded at 148 ± 3.39 mg.L^−1^, higher than the recommended value of NWQS (1 mg.L^−1^). According to El-Batrawy *et al*. (2018), high level of Fe in the water column can be associated with eutrophication. Due to the respiration and the decomposition of aquatic plants, CO_2_ is released into water and decreases water pH. Low pH will favor the leeching process of Fe in the sediment into the water column. Furthermore, the soil in Peninsular Malaysia consists of laterite soil, which is rich in Fe, and the soil is known to act as a reservoir for trace elements (Shuhaimi-Othman *et al*. 2010). Fe in the sediment can be released into the water and bioaccumulate in the fish. Nevertheless, Fe’s high concentration is the least concern because Fe toxicity is rare and Fe deficiency is much more prevalent in humans.

Mn is another essential trace metal element that plays a vital role as a cofactor in many enzymatic pathways in both human and aquatic organisms (Pan *et al*. 2008; Lee *et al*. 2010). Even though it is low in toxicity, high level of Mn will cause intoxication, reproduction impairment, neurological problems, and inflammation of the respiratory organs, such as lungs (Mahboob *et al*. 2014; Korkmaz *et al*. 2017). According to the Malaysian Food Act (1983) and Regulations (1985), and WHO (1985), Mn’s permissible levels are set at 0.3 mg.kg^−1^ and 100 mg.kg^−1^, respectively. Based on these levels, the Mn level in the muscle of *O. niloticus* (0.04 mg.kg^−1^) in the study was below the permissible level. A comparison of the Mn level with the literature shows that the trace element was within the reported range for Mn in other fish species in Malaysia, such as 2.3 mg.kg^−1^–2.8 mg.kg^−1^ in the fish from former mining pond in Perak (Azlan *et al*. 2017), and 0.012 mg.kg^−1^– 0.028 mg.kg^−1^ in fish samples from Galas river (Zarith Sufiani & Mohd Yusoff 2015).

Pb is a toxic non-essential metal of which the toxicity is dependent on its chemical form. The organo-Pb compounds are known to be more toxic than the inorganic form (Bosch *et al*. 2016). The early manifestation of Pb intoxication in animals is anemia because most of the Pb absorbed by animals is taken by the red blood cells. The Pb interferes with the synthesis of the haem group, which is the key component in hemoglobin for oxygen transportation. Pb also changes the structural integrity of fish gills due to the deposition of mucus produced under hypoxic conditions (Mahboob *et al*. 2014). Pb also affects the nervous system, embryonic development and causes kidney failure. Like other trace elements, Pb accumulates in high concentrations in liver, followed by kidney and muscle. The concentration of Pb in this study was much lower than the permitted value of Pb according to the Malaysian Food Act (1983) and Regulations (1985), which is set at 2.0 mg.kg^−1^. The accumulation levels for Pb in this study were found to be within the reported range for Pb in the muscle tissue of fish in Malaysia (0.010 mg.kg^−1^– 0.078 mg.kg^−1^) (Taweel *et al*. 2013b; Yaakub *et al*. 2017; Ishak *et al*. 2020; Rosli *et al*. 2021).

Zn is considered an essential trace element required for the function of more than 300 enzymatic reactions in many physiological processes in living organisms. Zn deficiency may lead to slow growth, impaired immunology, and skin problems. However, if the level of Zn reaches 50 mg.kg^−1^ in the muscle, it will cause toxicity, such as diarrhea, nausea and acute stomach pain (Mahboob *et al*. 2014). Higher Zn bioaccumulation in fish can possibly be due to the higher content of Zn in the surrounding environment, either in dissolved or suspended form. The Zn content (muscle tissue) in the literature has been recorded at 0.26 mg.kg^−1^– 6.75 mg.kg^−1^ and the value reported for Zn in this study was within the range mentioned in the previous studies on freshwater fish in Lake Chini, Lake Beranang and Galas River (Ahmad *et al*. 2008; Zarith Sufiani & Mohd Yusoff, 2015; Azlan *et al*. 2017). In this study, the Zn content in all parts of the fish was found to be lower than the permitted value set by the Malaysia Food Act (1983) and Regulations (1985) at 100 mg.kg^−1^. This can also be caused by the leaching of paints containing Zn, which is used to prevent the oxidation of boat (Satapathy & Panda 2017).

Human health assessment on the consumption of *O. niloticus* as assessed by the non-carcinogenic (EDI, THQ and HI) indexes established no potential health risk to the consumers. Many studies in *O. niloticus* in this country corroborate this finding (Taweel *et al*. 2013a; Kah *et al*. 2015; Kok & Ke 2017; Ishak *et al*. 2020). In this study, the CRs of Pb and _i_As in *O. niloticus* were found within the acceptable range of 10^−6^ ≤ CR ≤ 10^−4^, reflecting the non-likelihood of getting cancer in the human lifespan due to the exposure of Pb and _i_As through the consumption of the fish. This is similar to the report by Kah *et al*. (2015) on the CR of _i_As from aquaculture ponds in Jelebu, Negeri Sembilan. In other region, Arisekar *et al*. (2020) recorded higher value of CR for As, which ranged from 7.76 × 10^−6^ – 8.18 × 10^−4^ in *O. niloticus* collected from the Thamirabarani River, India. There is limited information on the CR of Pb in *O.niloticus* from this country. However, CR of Pb in other region reported by Kortei *et al*. (2020) showed higher value of CR for Pb in *O. niloticus* from Ankobrah and Pra Basin, Ghana, which ranged from 3.3 × 10^−3^. A study conducted by Ullah *et al*. (2017) on eight fish species, including *O. niloticus* in Bangladesh’s market, also reported higher value of CR at 4.2 × 10^−6^.

## CONCLUSION

This study provides the first preliminary report on the concentration of trace elements in *O. niloticus* in BML. The liver accumulated the highest concentration of trace elements compared to the gill and the muscle tissue. The estimation of non-carcinogenic risk reflected no significant health risk due to the consumption of the fish. The carcinogenic risk also showed no potential of developing cancer due to the consumption of the fish. Constant monitoring and assessment are needed in order to conserve and to manage the fish resources in the lake. It is suggested that future research to be conducted on other species of fish in the lake and the influence of temporal and spatial variations on the bioaccumulation of trace elements in the water and sediment for more comprehensive information on the safety status of fish in the BML.

## Figures and Tables

**Table 1:** The results of trace elements concentrations in certified reference material (DORM 4, fish protein), *n* = 2.

Trace elements	Certified value (mg.kg^−l^)	Found value (mg.kg^−1^)	Mean recovery (%)
As	6.87 ± 0.44	7.07 ± 0.08	97.2
Cd	0.29 ± 0.02	0.31 ± 0.01	94.6
Cr	1.87 ± 0.18	1.87 ± 0.01	100.0
Cu	15.7 ± 0.46	15.49 ± 0.19	101.4
Fe	343 ± 20.0	340.44 ± 1.17	100.8
Mn	3.17 ± 0.26	3.03 ± 0.03	104.7
Ni	1.34 ± 0.14	1.25 ± 0.03	107.0
Pb	0.40 ± 0.26	0.45 ± 0.00	89.4
Zn	51.6 ± 2.80	52.56 ± 0.44	100.1

**Table 2:** Trace elements contents (mg.kg^−1^ w.w) in three different parts of *O. niloticus* from Bukit Merah Lake.

Fish part	As	Cu	Cr	Fe	Mn	Pb	Zn
Gills	0.4 ± 0.01	0.14 ± 0.0	0.05 ± 0.01	16.8 ± 0.38	1.8 ± 0.08	0.57 ± 0.01	2.44 ± 0.26
Muscle	0.06 ± 0.01	0.06 ± 0.0	0.02 ± 0.0	0.73 ± 0.07	0.04 ± 0.0	0.10 ± 0.03	0.53 ± 0.01
Liver	0.7 ± 0.01	41.47 ± 0.60	BDL	224.21 ± 5.05	0.35 ± 0.0	0.21 ± 0.00	1.89 ± 0.02
WHO	2	30	0.6	0.5	100	1.5	30
MFA	1	30	0.2	0.5	0.3	2.0	100

*Notes*: BDL = below detection level (Cd and Ni, were below detection level in all parts of the fish); WHO 1985 = World Health Organisation; MFA = Malaysian Food Act (1983) and Regulation (1985)

**Table 3:** Assessment of health indexes in *O. niloticus* in Bukit Merah Lake.

Heavy metals	EDI (mg.kg^−1^)	Recommended value (mg.kg^−1^)	THQ	HI	CR
As	2.63 × 10^−6^	0.13*	8.75 × 10^−3^		3.94 × 10^−6^
Cr	5.25 × 10^−5^	0.20*	3.50 × 10^−5^		
Cu	1.58 × 10^−4^	0.50*	3.94 × 10^−3^		
Fe	1.92 × 10^−3^	0.80*	2.74 × 10^−3^	1.00 × 10^−1^	
Mn	1.05 × 10^−4^	NA	7.50 × 10^−4^		
Pb	2.63 × 10^−4^	0.21*	7.50 × 10^−2^		2.23 × 10^−6^
Zn	1.39 × 10^−3^	0.3–1*	4.64 × 10^−3^		

*Notes*:

* Joint FAO/WHO Expert Committee on Food Additives (2009); EDI = Estimated daily intake; THQ = Target hazard Quotient; HI= Hazardous Index; CR= Carcinogenic risk; NA = not available
